# Intracochlear Bleeding Enhances Cochlear Fibrosis and Ossification: An Animal Study

**DOI:** 10.1371/journal.pone.0136617

**Published:** 2015-08-26

**Authors:** Kyeung A. Ryu, Ah-Ra Lyu, Heesung Park, Jin Woong Choi, Gang Min Hur, Yong-Ho Park

**Affiliations:** 1 Department of Otolaryngology-Head and Neck Surgery, College of Medicine, Chungnam National University, Daejeon, Republic of Korea; 2 Department of Pharmacology, College of Medicine, Chungnam National University, Daejeon, Republic of Korea; 3 Brain Research Institute, College of Medicine, Chungnam National University, Daejeon, Republic of Korea; National Cheng Kung University, TAIWAN

## Abstract

The aim of this study was to investigate the effects of intracochlear bleeding during cochleostomy on cochlear inflammatory response and residual hearing in a guinea pig animal model. Auditory brainstem response threshold shifts were greater in blood injected ears (*p*<0.05). Interleukin-1β, interleukin-10, tumor necrosis factor-α and nitric oxide synthase 2, cytokines that are related to early stage inflammation, were significantly increased in blood injected ears compared to normal and cochleostomy only ears at 1 day after surgery; with the increased IL-1β being sustained until 3 days after the surgery (*p*<0.05). Hair cells were more severely damaged in blood injected ears than in cochleostomy only ears. Histopathologic examination revealed more extensive fibrosis and ossification in blood injected ears than cochleostomy only ears. These results show that intracochlear bleeding enhanced cochlear inflammation resulting in increased fibrosis and ossification in an experimental animal model.

## Introduction

Cochlear implant surgery is a widely used surgical procedure done to restore hearing in patients with severe and profound hearing loss. Preservation of residual hearing is an important prerequisite in achieving maximal benefit from cochlear implant surgery since residual hearing loss would diminish the effectiveness of a cochlear implant. In most patients, residual hearing is immediately preserved after cochlear implantation especially with the development of soft surgery or the round window approach[1, 2]. Unfortunately, it has been reported that approximately one third of cochlear implant recipients would slowly lose their residual hearing over the next few months[3–6]. In a postmortem human temporal bone study of cochlear implantation, the auditory threshold levels were increased with greater amount of intracochlear fibrosis and ossification[7–9]. The ensuing fibrosis and ossification in the cochlea may account for the delayed hearing loss following cochlear implantation. Although the reasons for this phenomenon are not well understood, the tissue response to implantation may be implicated[10–14].

Cochleostomy, in itself, may induce intracochlear inflammation due to the bone dust produced during the procedure in addition to the trauma of electrode insertion. However, there may be other factors which could induce intracochlear inflammation that warrant further investigation. Within the temporal bone, the cochlea is located inside a bony structure which is covered by mucosa that has microvasculatures. Intraoperative bleeding commonly occur from this mucosa and could easily enter the cochlea during surgery. This intracochlear blood contamination may be a source of intracochlear inflammation, but the effect of intracochlear bleeding is not well known. In this study, we aimed to investigate the effect of intracochlear bleeding during cochleostomy on intracochlear inflammation.

## Materials and Methods

### Animals

All animal experiments were approved by the Chungnam National University, Committee of the Animal Experiment (CNU00322, CNU00499). Twenty-nine male albino guinea pigs, weighing 250-300g each, with normal hearing prior to surgery were enrolled in this study. Twenty three animals were used for the experimental group with cochleostomy (see below): the left ears underwent cochleostomy with blood injection into the scala tympani and the right ears underwent cochleostomy only (control ear). The experimental animals were used for the time point studies (5 animals for 1, 3, and 14 days, 4 animals for 7 and 60 days). The other six animals were used as normal controls for real time polymerase chain reactions (N = 3) and histopathologic studies (N = 3).

### Surgical procedure for cochleostomy and intracochlear blood injection

Before surgery, the animals were anesthetized with intramuscular injection of combination of tiletamine HCl and zolazepam HCl 40 mg/kg (Zoletil, Virbac Animal Health, Carros, France) and xylazine 10 mg/kg (Rompun, Bayer Animal Health, Monheim, Germany). In addition, 0.5 ml of 1% lidocaine HCl was injected subcutaneously in the postauricular area for local anesthesia. The animals were placed in a prone position on a thermoregulated heated pad. After a retroauricular incision, blood was taken from a neighboring blood vessel using an insulin syringe. The temporal bone was exposed and opened to visualize the round window membrane. A small cochleostomy was made bilaterally in the bone near the round window with a sharp pick. Using a microcannula connected to the tip of a 30 gauge needle and Hamilton syringe (Hamilton Company, Reno, NV), 5μl of blood was injected into the scala tympani through the left cochleostomy site using an infusion pump for 2 minutes. The cochleostomy site and bulla were then sealed with tissue adhesive (Durelon, 3M ESPE, Germany) and carboxylate cement (Durelon, 3M ESPE, Germany). The skin incision was closed in two layers. Afterwards, the animals were allowed to recover from anesthesia, and their pain was controlled with carprofen (Rimadyl, 4mg/kg, subcutaneously, Pfizer, NY, USA). There were no mortalities in animals used in this set of experiments. Morbidity was limited to signs that are typical after cochleostomy, including unsteadiness and occasional head tilt. These resolved within a few days and did not worsen.

### Auditory brainstem response

Auditory brainstem response (ABR) thresholds from 4 to 32 kHz, and click sound were obtained separately from both ears. The ABRs were recorded prior to surgery and just after surgery. TDT System-3 (Tucker Davies Technologies, Gainseville, FL, USA) hardware and software were used to obtain the ABRs. The stimuli were computer generated tone pips. Subcutaneous needle electrodes were placed around the skull vertex and both infra-auricular area. Tone bursts, with duration of 4 ms and a rise-fall time of 1 ms at frequencies of 4, 8, 16, 32 kHz, and clicks were used. The sound intensity was varied by 10-dB intervals for the tone-burst sounds and by 5 dB intervals for the click sounds near the threshold. The contralateral ear was not masked because the stimuli were transmitted through a sealed earphone. The waveforms were analyzed using a custom program (BioSig RP, ver. 4.4.1) with the researcher blinded to the treatment group. Threshold was defined as the lowest stimulus intensity to evoke a wave III response greater than 0.2 mV.

Further ABR threshold measurements were done at 7 days and 2 months after the operation. The differences in ABR thresholds were averaged across the frequency range for each cochlea to yield their individual mean rise in ABR threshold. Threshold shift was defined as the difference between preoperative and one of the postoperative values. A positive threshold shift indicated an elevation of the auditory threshold.

### Quantitative real time polymerase chain reaction

Corresponding animals were sacrificed at either 1 or 3 days after the surgical procedures and quantitative real time polymerase chain reactions (qRT-PCR) were conducted to look for evidence of inflammation. Interleukin-1β (IL-1β), interleukin-10 (IL-10), tumor necrosis factor-α (TNF-α) and nitric oxide synthase 2 (NOS 2) were measured and used as indicators of inflammatory response.

Dissected cochleae were ground in 1 ml of TRIZOL reagent (Invitrogen, Carlsbad, CA, USA), 200μl of chloroform was added, and then centrifuged at 13000 rpm for 15 minutes. About 450 μl of supernatant was transferred to a fresh tube and the same amount of isopropanol was added, shaken for 5 minutes, and centrifuged at 13000 rpm for 15 minutes. The resulting pellet was resuspended in 1 ml of 80% ethanol (in DEPC-treated water) and centrifuged at 13,000 rpm for 15 minutes. The same procedure was performed one more time and the pellet was then washed with 100% ethanol repeatedly. RNA was dissolved in 20 ul of RNase-free water. The purified RNA was quantified using Nano drop (NanoDrop Technologies Inc., Wilmington, DE, USA) by measuring UV absorbance of 260 nm. A total 13 μl of RNA (2 μg each) with oligo-dT primer and DEPC-treated water was pre-denatured for 10 minutes at 65°C and 5x reaction buffer 4 μl, dNTP 2 μl, RNase inhibitor 0.5 μl, RTase 0.5 μl were added and reverse transcribed for 1 hour at 50°C and 5 minutes at 85°C with the cDNA Synthesis Kit (Roche, IN, USA). The real-time reverse transcription process was performed according to the manufacturer’s procedure with SYBRgreen (Invitrogen, Grand Island, NY, USA). Comparative quantification of IL-1β, IL-10, TNF-α, and NOS 2 mRNA was obtained by comparative cycle of the threshold method. The quantitative RT-PCR was performed 3 times for each sample. The details of primers used in the polymerase chain reaction to detect IL-1β, IL-10, TNF-α, and NOS 2 are presented in Table 1.

**Table 1:** Primers used in polymerase chain reaction to detect IL-1β, IL-10, TNF-α, and NOS 2.

Primer name		Primer
GAPDH	Forward	5'-GCCCTCAATGACCACTTTGT-3'
	Reverse	5'-TGCTGTAGCCGAACTCATTG-3'
IL-1β	Forward	5'-TCCCTGTGAAAACAAGAGCA-3'
	Reverse	5'-CGCCTTTCTCTTGGAGCTTA-3'
IL-10	Forward	5'-ACACCCAGTCTGAGGACAGC-3'
	Reverse	5'-AAAGTCTTCACCCTGCCAAA-3'
TNF-α	Forward	5'-ATCAAGAGTCCCTGCCAGAA-3'
	Reverse	5'-CTCCCAGGTAGATGGGTTCA-3'
NOS2	Forward	5'-CCCTCTTCGTGCTGAAAAAG-3'
	Reverse	5'-GTCATGAGCAAAGGCACAGA-3'

IL-1β: Interleukin-1 beta, IL-10: Interleukin-10, TNF-α: tumor necrosis factor-alpha, NOS 2: nitric oxide synthase 2.

### Tissue preparation and Immunohistochemistry

The animals were sacrificed 2 weeks after surgery and cochlear tissues were obtained to assess survival of hair cells and nerve fibers. Tissues were fixed in 4% paraformaldehyde in PBS for 1 hour at room temperature. After removal of the cochlear bony walls and lateral wall tissues, the remaining cochlear tissues were prepared for immunostaining. Tissues were permeated with 0.3% Triton X-100 (Sigma–Aldrich Co., St. Louis, MO) for 10 minutes, blocked in 5% normal goat serum (Vector Laboratories, Inc., Burlingame, CA) for 30 minutes and were then incubated with rabbit anti-myosin VIIa primary antibody (Proteus BioSciences, Inc., Ramona, CA) and mouse anti-NF200 primary antibody (Novus Biologicals, Littleton, CO) at a concentration of 1:200 in blocking solution overnight at 4°C. After rinsing in PBS for 10 minutes, the tissues were incubated with the corresponding AlexaFluor 594 goat anti-rabbit secondary antibody (Molecular Probes, Eugene, OR) or AlexaFluor 488 goat anti-mouse secondary antibody (Molecular Probes, Eugene, OR) at a concentration of 1:200 in PBS for 30 minutes. Tissues were then rinsed in PBS. Only tissues stained with myosin VIIa were double labeled with AlexaFluor 488 phalloidin (Molecular Probes, Eugene, OR) at a concentration of 1:500 for 30 minutes. After rinsing in PBS for 10 minutes, specimens were further dissected to separate individual cochlear turns and mounted on glass slides using CrystalMount (Biomeda, Foster City, CA). The specimens were observed using an epifluorescence microscope (Zeiss Axio Scope A1; Zeiss, Germany) with digital camera and the surviving hair cells were counted in each 100 μm.

**Fig 1:**
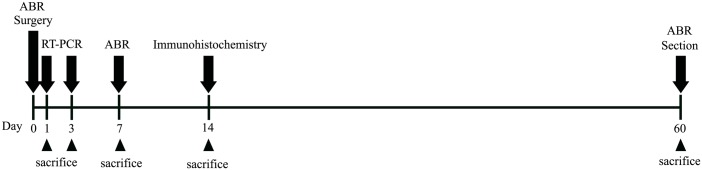
Schematic time-line of experiments. ABR thresholds were measured at prior to surgery, just after surgery, 7 days and 2 months after surgery. qRT-PCRs were conducted at 1 and 3 days after surgery for the evaluation of acute inflammatory responses. Assessment of hair cells and nerve fiber survivals were evaluated at 2 weeks after surgery. Intracochlear histopathologic changes were evaluated at 2 months after surgery.

To assess intracochlear fibrosis and ossification, both cochleae were harvested from the animals 2 months after the operation. The harvested samples were placed in 4% paraformaldehyde in PBS for 2 hours, decalcified in EDTA (ethylene diamine tetraacetic acid, 5% Nitric acid) for 3 weeks, paraffin embedded, sectioned in the mid-modiolar plane at a thickness of 5 μm and stained with hematoxylin and eosin. The stained tissue sections were examined and representative fields photographed using a light microscope (Olympus BX51; Olympus, Tokyo, Japan). All histologic sections were examined for evidence of intracochlear fibrosis and new bone formation. The timeline for all experiments are shown in Fig 1.

### Image processing and statistical analysis

Adjustment of image contrast, superimposition of images, and colorization of monochrome fluorescence images were performed using Adobe Photoshop (version 7.0). Statistical analysis was performed with Graphpad Prism (version 3.02, San Diego, CA, USA). ABR threshold shift and the levels of inflammatory cytokine data taken before and after the surgery in each group were compared using analysis of variance (ANOVA). The hair cell survival between groups were compared using Student t-test. *p* values of < 0.05 were considered significant.

## Results

### ABR threshold shifts

**Fig 2:**
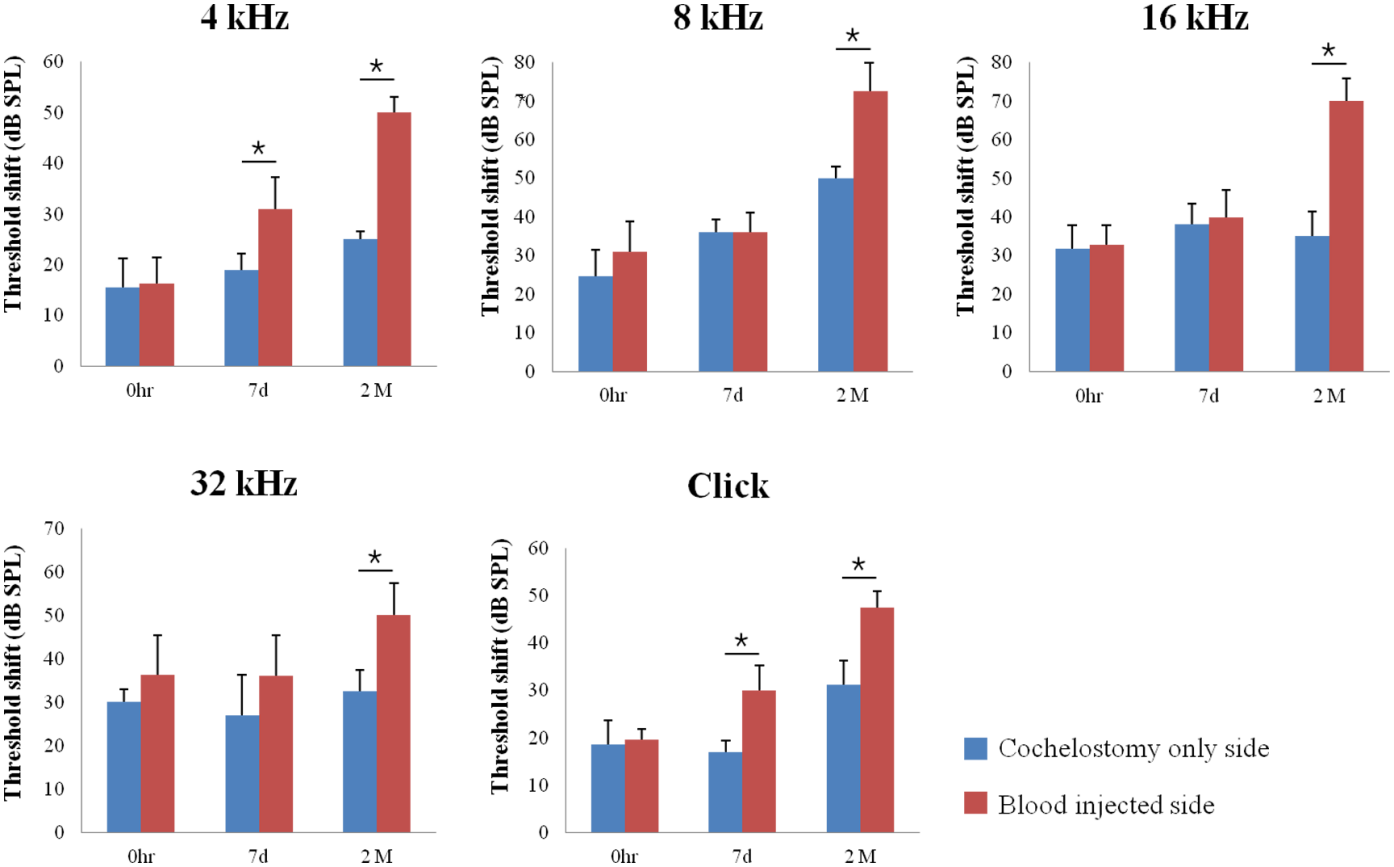
Auditory brainstem response threshold shifts at just after surgery, 7 days and 2 months after surgery. ABR threshold shifts were greater in blood injected ears compared to cochleostomy only ears. Asterisk indicates p < 0.05.

To evaluate the hearing threshold shifts in each group after surgery, ABR threshold from 4, 8, 16, 32 kHz, and click sound were recorded prior to surgery, just after surgery, at 7 days and 2 months after surgery. ABR threshold shifts were greater in blood injected ears compared to control ears. The differences were significant with click and 4 kHz at 7 days after surgery, and all measured frequencies at 2 months after surgery (Fig 2.)(*p*<0.05). This suggested that the ensuing cochlear damage was more severe in blood injected ears.

### Inflammatory cytokines changes

**Fig 3:**
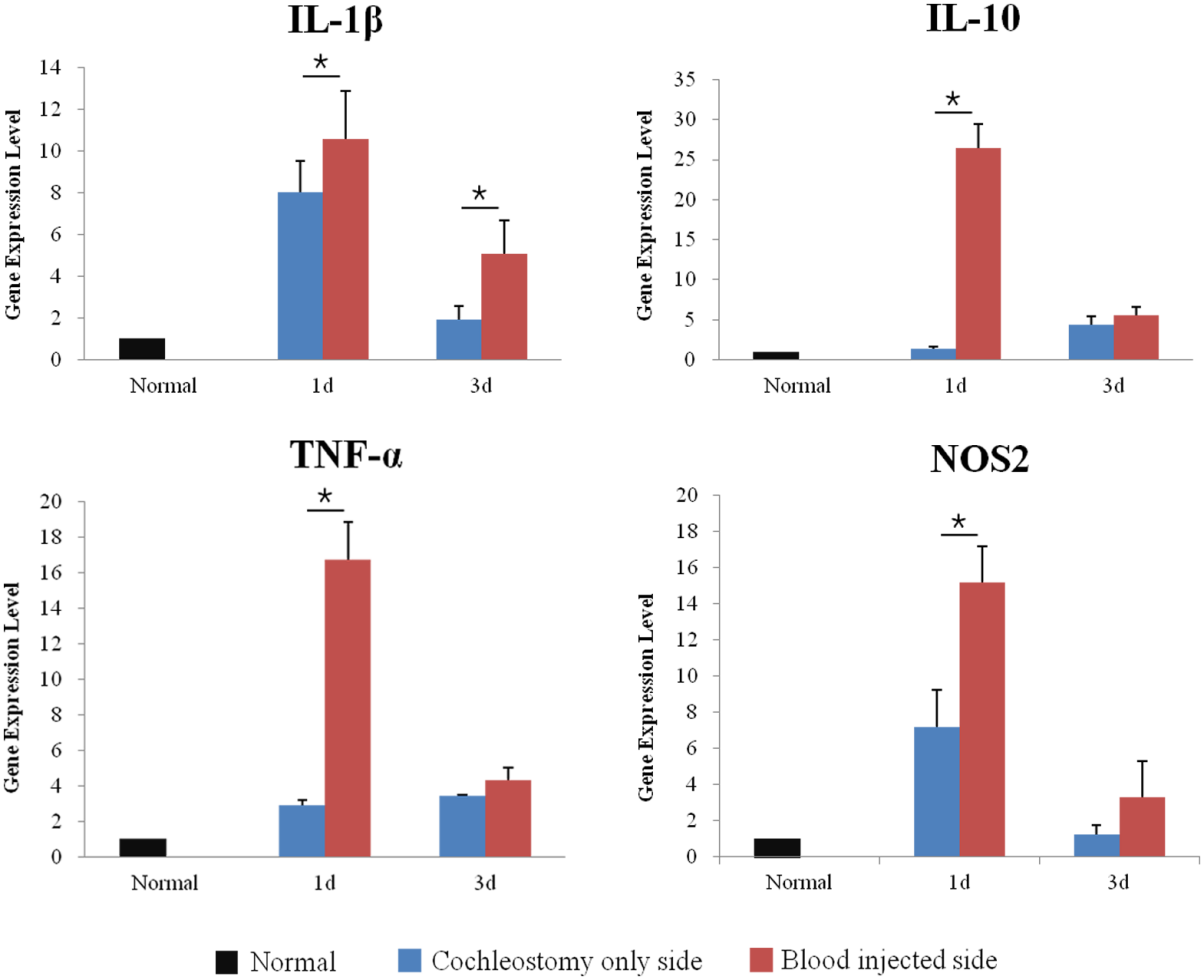
Quantitative real time polymerase chain reactions at 1 and 3 days after surgery. IL-1β, IL-10, TNF-α, and NOS 2 were significantly increased in blood injected ears compared to normal and control ears at 1 day, and the increased IL-1β was sustained until 3 days after surgery. Asterisk indicates p < 0.05.

To compare the inflammatory responses between groups, the animals were sacrificed at either 1 day or 3 days after surgery and qRT-PCR for IL-1β, IL-10, TNF-α, and NOS 2 were conducted. IL-1β, IL-10, TNF-α, and NOS 2 were significantly increased in blood injected ears compared to the normal and control ears at 1 day after surgery. The increase in IL-1β was sustained until 3 days after surgery (Fig 3) (*p*<0.05). This suggested that blood injected ears had more severe inflammation and it occurred during the early stage.

### Hair cell survival

**Fig 4:**
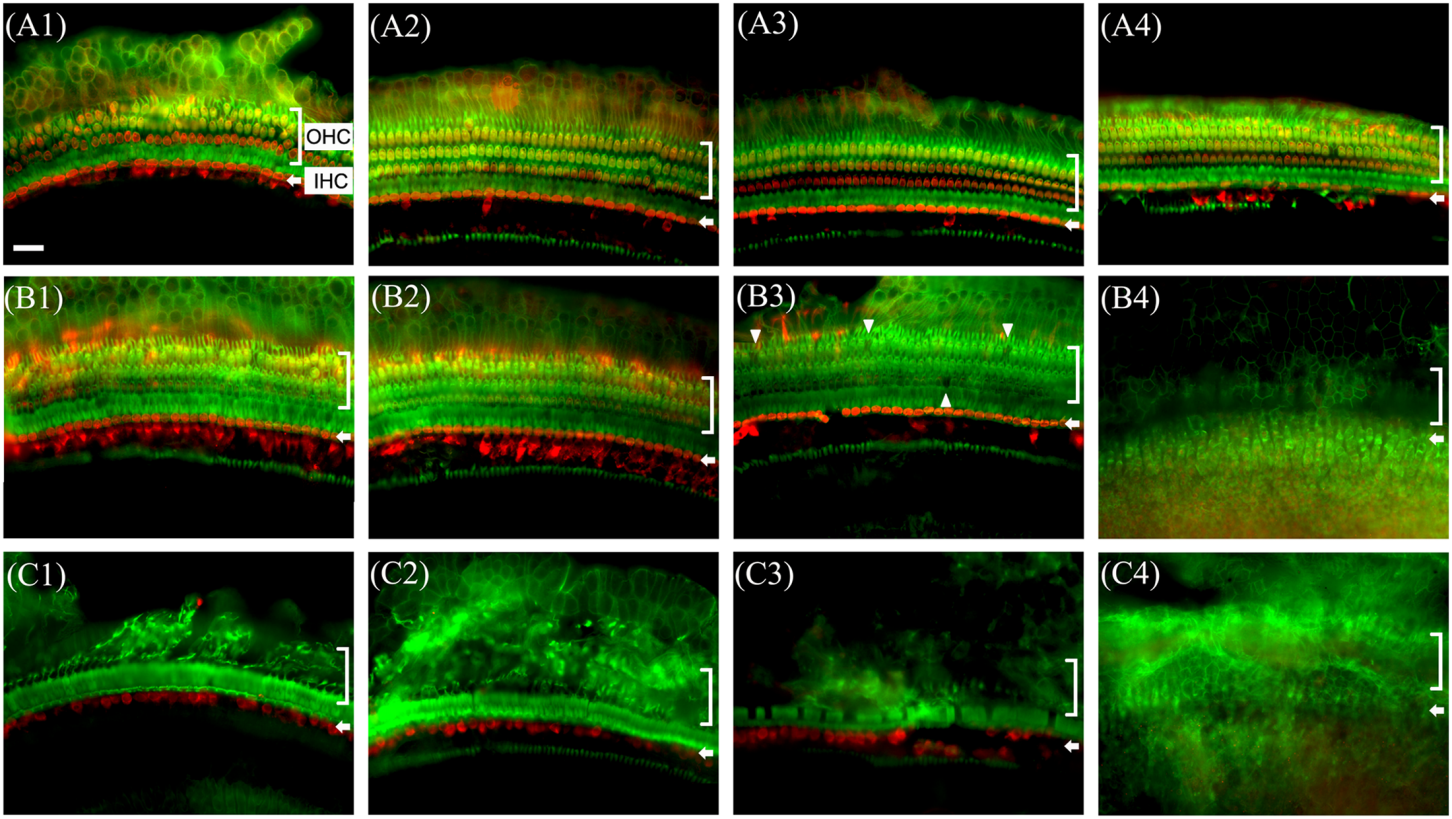
Whole-mounts of the auditory epithelium in normal (A1, A2, A3 and A4), cochleostomy only (B1, B2, B3 and B4) and blood injected ear (C1, C2, C3 and C4) at 2 weeks after surgery stained for myosin VIIa (red) for hair cells and rhodamine phalloidin (green) for actin and photographed with epifluorescence. Hair cells loss was more severe in blood injected ear (C1~C4) than control ear (B1~B4). In blood injected ear, severe outer hair cell loss is observed from the basal turn up to the apex (C1~C4). A1, B1 and C1: Apical turn, A2, B2 and C2: 3rd turn, A3, B3 and C3: 2nd turn, A4, B4 and C4: basal turn, OHC: outer hair cell, IHC: inner hair cell. Scale bar = 30 μm.

To assess survival of hair cells in the cochleae, the animals were sacrificed at 14 days after surgery and whole mounts of the auditory epithelium were stained with antibody against myosin VIIa (red, hair cells) and rhodamine phalloidin (green, actin) for epifluorescence. Almost all the outer hair cells were destroyed (Fig 4C) in the blood injected ears compared to control ear (Fig 4B) and this finding continued throughout the entire cochlea, upto the apical turn (Fig 4C1, 4C2, 4C3 and 4C4). In control ears, the destruction of hair cells was observed only in the basal turn and surviving hair cells were observed in the upper turns (Fig 4B1, 4B2, 4B3 and 4B4).

### Nerve fiber survival

**Fig 5:**
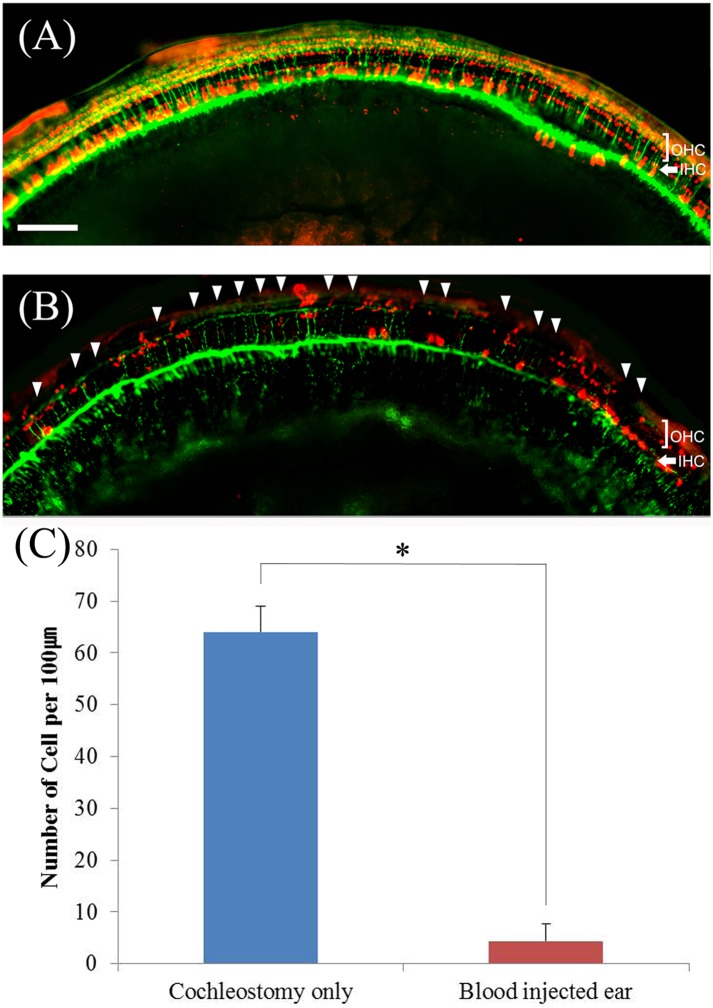
Whole-mounts of the upper second turn of auditory epithelium in cochleostomy only (A) and blood injected ear (B) at 2 weeks after surgery stained for myosin VIIa (red, hair cells) and NF200 (green, nerve fibers) and photographed with epifluorescence. Hair cell loss (arrow head) and nerve degeneration were more severe in blood injected ear (B) than control ear (A). Hair cell counts showed that significant hair cell loss in blood injected ear (C). OHC: outer hair cell, IHC: inner hair cell. Scale bar = 80 μm.

To assess survival of nerve fibers in the cochleae, the animals were sacrificed at 14 days after surgery and whole mounts of the auditory epithelium were stained with antibody against myosin VIIa (red) and NF-200 (green) for epifluorescence. Intact nerve fibers with surviving hair cells were observed (Fig 5A) in control ears but the blood injected ears showed markedly decreased nerve fibers with severe destruction of hair cells (Fig 5B). Hair cell counts also showed that severe hair cell loss were observed in blood injected ears compared to control ear (Fig 5C) (*p*<0.05).

### Intracochlear histopathologic changes

**Fig 6:**
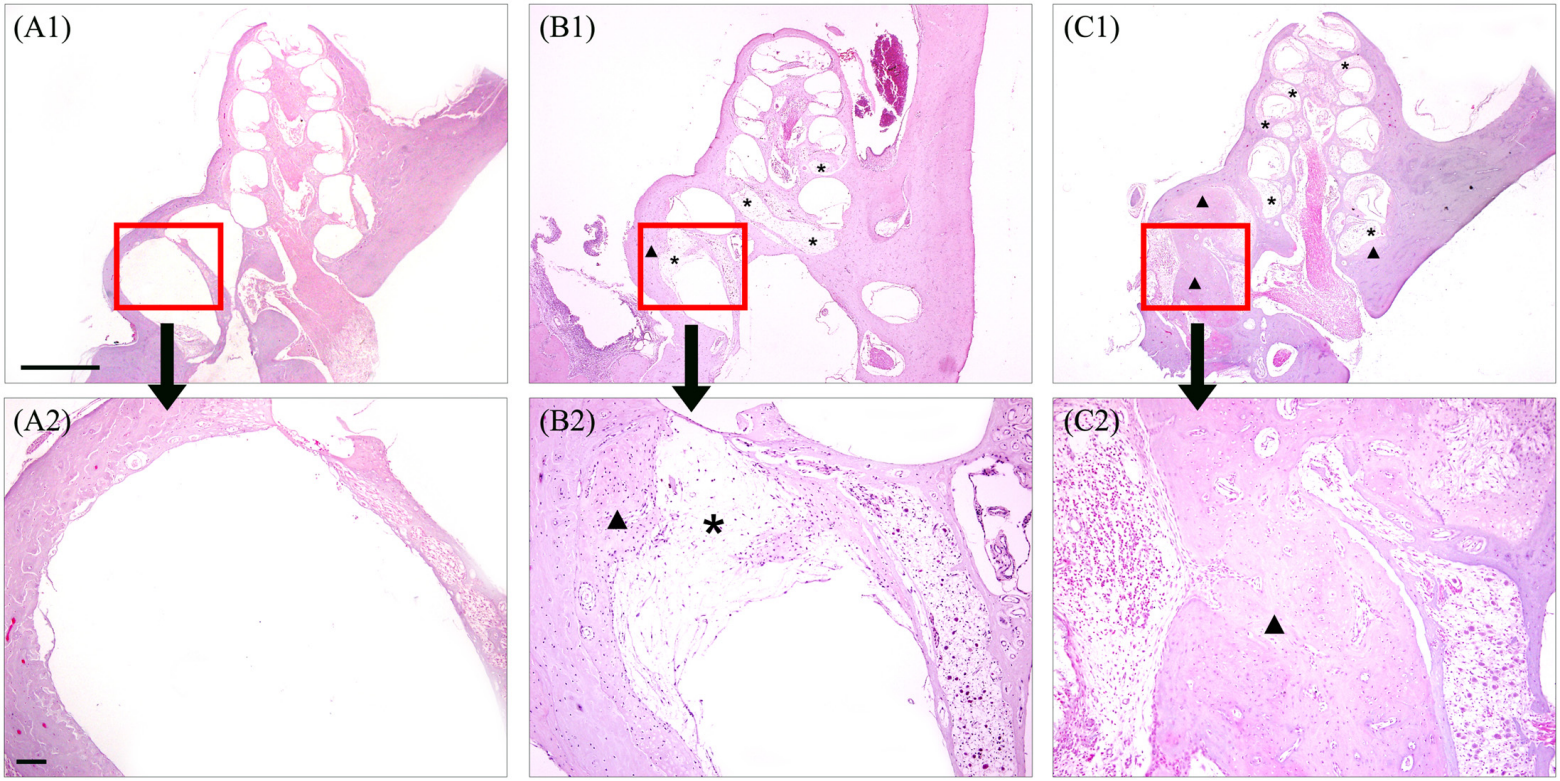
Sectional histopathologic findings of cochlea in normal (A), cochleostomy only ear (B) and blood injected ear (C) at 2 months after surgery. More extensive fibrosis (asterisk) and ossification (arrow head) were observed in blood injected ear (C1 and C2) than control ear (B1 and B2). Scale bar = 500 μm in A1, B1 and C1 and 50 μm in A2, B2 and C2.

For evaluation of intracochlear fibrosis and ossification, animals were sacrificed at 2 months after surgery and mid-modiolar sections were prepared. Comparison of tissue growth or fibrosis and new bone formation between the blood injected ears and control ears was made. In the control ears, fibrosis and mild ossification were observed in the basal area and second turn of the cochlea (Fig 6B1 and 6B2). In the blood injected ears, more extensive fibrosis throughout the entire cochlea was observed in the scala tympani and scala vestibuli with more severe ossification in the basal turn (Fig 6C1 and 6C2).

## Discussion

To develop strategies for the preservation of residual hearing following cochlear implantation, researchers continue to investigate the pathophysiology of hearing loss after cochlear implantation. The suggested etiologies and pathogenesis for hearing loss after cochlear implantation included; direct mechanical trauma from the implanted electrode and hydraulic damage following the insertion of volume displacing cochlear implant within the scala tympani[10, 15–18].

A number of investigators have examined these suggested etiologies and pathogenesis in post mortem studies of human cochlear implant recipients[7, 9, 12, 15, 19, 20], cadaveric temporal bones[16, 18, 21–24] and animal studies of cochlear implantation[13, 25–28]. In the human temporal bone study, damage from insertion of the long electrode was located mainly at the most anterior part of the basal turn in 16 temporal bones with cochlear implants[7]. Although implantation did not affect the spiral ganglion cell count on the implanted ears[9, 20], the inserted electrode could have scratched the scala tympani lateral wall, disrupted the basilar membrane and Reissner’s membrane, and be positioned in the scala vestibuli[10, 29, 30]. The histopathologic findings also showed cochlear fibrosis and ossification in both human and animal cochlear implantation model[7, 9, 19, 23, 28, 31]. These studies supported that delayed intracochlear fibrosis and ossification due to the trauma of electrode insertion may cause residual hearing loss and reduce the beneficial effect of the cochlear implant. The histopathologic findings in our study showed that intracochlear fibrosis and ossification appeared 2 months after the cochleostomy with blood injection and these intracochlear changes coincided well with other temporal bone studies. The current study showed that the fibrosis and ossifications usually affected the scala tympani at the basal turn of the cochlea where the cochlear implant electrode was positioned and seemed to extend toward the upper turns and scala vestibuli.

The soft surgery through the round window approach[1, 2, 32, 33] and development of atraumatic electrodes could decrease electrode insertion trauma and reduce residual hearing loss[21, 23, 24, 34]. More recently, perioperative dexamethasone[35–38] and development of dexamethasone eluting electrode have been considered[39–42] since reports claimed that dexamethasone could prevent hearing loss[35–38, 43, 44], reduce tissue response around the electrode[38, 41]and may have an effect on the normalization of homeostasis in the cochlea after cochlear implantation[45]. In this study, an electrode or electrode equivalent was not included in the experimental procedure because our intention was just to demonstrate and prove the effect of intracochlear bleeding. The results revealed that, even in the absence of an electrode, inflammation occurred and resulted in extensive fibrosis and ossification in the blood injected ears. Employing different surgical procedures and electrodes may affect the above results. Further studies using an electrode with anti-inflammatory agent may be more useful and relevant.

Although inflammatory responses after electrode insertion with mechanical trauma may be the main reason for cochlear fibrosis and ossification, the precise etiology is not yet known. A recent report claimed that injury or occlusion of blood vessels associated with the scala tympani may adversely affect inner ear function and contribute to hearing loss after electrode insertion[46]. The effect of intracochlear bleeding as a cause of residual hearing loss after cochlear implantation surgery is, however, not yet well known. In the lungs, diffuse pulmonary fibrosis and ossification has been associated with prior diffuse alveolar hemorrhage[47–49]. Alveolar bleeding is responsible for interstitial metallic deposition that attracts calcium salts and multinucleated giant cells[48]. Blood, in itself, has lots of inflammatory components. We thought that intracochlear bleeding could also contribute to intracochlear fibrosis and ossification.

To prove the hypothesis, we studied the effect of intracochlear bleeding on cochlear inflammation. Results show that the intracochlear blood injected ears showed more extensive fibrosis and ossification in the cochlea compared to control ears. These results coincided with more severe hair cell loss up to the apical turn of the cochlea and greater ABR threshold shifts in blood injected ears compared to control ears. To support these results, we conducted qRT-PCR for inflammatory markers in each group. The results showed that IL-1β, IL-10, TNF-α, and NOS 2 were significantly increased in blood injected ears on the first day after surgery compared to control ears. It seems that a more severe inflammation occurred in blood injected ears during the early stage.

A limited investigation on the effect of fibrosis within the cochlea resulting from inflammation on acoustic hearing has demonstrated some correlations between audiology and histology in animal studies[13]. This relationship has been more precisely modeled mathematically. Choi et al[14] used a one dimensional model to calculate the effect of increased dampening from scala tympani fibrosis in the basal one half of the cochlea on basilar membrane vibration. They demonstrated a substantial reduction in basilar membrane velocity at the apex of the cochlea with increased dampening due to fibrosis associated with cochlear implantation. Animal studies support the accuracy of this model by demonstrating that the degree of fibrosis within the basal scala tympani correlated with increased ABR threshold, particularly in cases where the basal fibrosis was adjacent to the basilar membrane. In such circumstances, it was found that ABR threshold to click stimulus was increased[13]. The authors of this study speculated that this is because of altered basilar membrane biomechanics with impaired propagation of sound energy along the basilar membrane. Even in the absence of fibrosis, the electrode abutting the basilar membrane, by itself, can cause some degree of dampening[13]. In our study, even if we did not check for basilar membrane vibration, the ABR threshold shifts were greater in blood injected ears at all measured frequencies at 2 months after the procedure. We believe that the histopathologic findings, which showed severe hair cell loss, extensive fibrosis and ossification in the blood injected ears may be directly associated with the more severe hearing loss in the blood injected ears.

## Conclusion

We found that more severe inflammatory responses occurred in intracochlear blood injected ears than in control ears. The more severe inflammatory responses may induce more severe hair cell loss, extensive fibrosis and new bone formation affecting the cochlea leading to residual hearing loss in intracochlear bleeding. Prevention of intracochlear bleeding during cochleostomy may be an important factor in reducing intracochlear inflammation and eventual residual hearing loss.
